# Miscellany

**Published:** 1899-09

**Authors:** 


### MISCELLANY.

THE recent storms in Porto Rico have been so disastrous to life and property that an appeal is made to the people of the United States to contribute to their relief. The Merchants' Association of New

York has appointed a relief committee, with Governor Roosevelt as chairman, that has sent out the following appeal:

*To the People of the State of New York:*

More than 100,000 people of Porto Rico are dependent upon the charity of this country. They have been in a moment reduced to complete destitution. Their homes have been swept away, their business prostrated, their occupations stopped. Thousands of families are without roofs, without clothing, without food. They have no means of subsistence or protection. The usual resources of the Island are paralysed. They cannot help themselves, and I appeal to the people of the great State of New York to lead in giving them the relief so urgently needed.

The calamity which has befallen the people of Porto Rico is one of the greatest disasters of modern times, and many thousands will die from exposure, disease and famine unless the generosity of our countrymen comes promptly and largely to their relief.

By request of the Secretary of War, the Merchants' Association has undertaken this work, and I appeal to all patriotic citizens to show to the suffering people of our new possessions that the extension of our flag over their territory is to be of immediate material as well as moral benefit to them.

Large amounts of money are necessary to purchase food, clothing and medical supplies immediately, which will be distributed under supervision of the U. S. Army officers.

Checks may be made payable to S. C. Mead, treasurer, Porto Rico Relief Committee, The Merchants' Association of New York, 346 Broadway, New York City.

THEODORE ROOSEVELT, Chairman.

---

STATE OF NEW YORK—CIVIL SERVICE EXAMINATIONS.—Open competitive examinations will soon be held in various cities throughout the state for the positions mentioned below; candidates having applications on file will be given ample notice of the time and place of examination most convenient to their places of residence.

Intending competitors must file application in the office of the commission at least five days before the examination. No one will be admitted to examination without the official notice (form 8) from the chief examiner of the commission.

The following examinations will be held on or about September 30, 1899: Apothecary, bookkeeper, clerk, regents' clerk, fireman, inspector of public works, matron, messenger, page, physician, storekeeper.

Examinations for stenographers and typewriters will be held during the week beginning October 9, 1899.

For application blank and detailed circular, address State Civil Service Commission, Albany, N. Y.

